# Differentiating between girls and boys in transition through smoking stages: A sex-specific growth mixture modeling

**DOI:** 10.34172/hpp.2021.25

**Published:** 2021-05-19

**Authors:** Nasrin Jafari, Asghar MohammadpourAsl, Mohammad Asghari-Jafarabadi

**Affiliations:** ^1^Department of Statistics and Epidemiology, Tabriz University of Medical Sciences, Tabriz, Iran; ^2^Center of the development of interdisciplinary research in Islamic sciences and health sciences, Tabriz University of Medical Sciences, Tabriz, Iran

**Keywords:** Sex-specific, Growth mixture models, Smoking stages, Adolescents, Transition

## Abstract

**Background:** Smoking is a complex process, and adolescents pass through a number of different stages on the way to become smokers and it is sex-dependent.

**Methods:** In this cohort study, the illustrative samples of 10th-grade students (2241 girls and 2956 boys) were assessed using a multistage sampling in Tabriz, Iran. The main variables of the study were smoking status, intention to start smoking, and smoking during the past week/ month, which were collected using a valid and reliable instrument. Sex-specific GMMs were fitted to assess the transition through smoking stages.

**Results:** GMMs lead in a 2-class optimal model: "Occasional/Intending smokers" and "Non-smokers". GMMs indicated that girls had lower levels of smoking status, intention to start smoking, smoking during the past week/month in both classes (significant and negative intercepts: -8.5 to -0.6). In addition, transitions toward higher levels of smoking status, intention to start smoking, smoking during the past week/month were observed in both classes for boys, but in the second class for girls (significant and positive slopes: 0.2 to 2.7).

**Conclusion:** This study highlighted the importance of stopping the initiation and avoiding transition through smoking stages with special sex-specific planning in the future.

## Introduction

Many studies reports the burden of smoking-related diseases and their mortality,^1^ the prevalence,^2-4^ theoretical models,^5^ and risk factors^6^ for smoking initiation in adolescents.

Nevertheless, smoking is a complex process, and adolescents pass through a number of different stages on the way to become smokers.^7^ Few studies, especially few longitudinal ones, have been conducted on the transition through smoking stages and the predictors.^7-11^ Determining the process of smoking stages and modeling for predictors is necessary to implement policies.

On the other hand, the smoking behavior is different between boys and girls,^12^ and the rate of smoking experimentation is sex-dependent. This rate is higher in boys than girls,^13^ and boys tend to initiate smoking earlier than girls.^13-15^ It was found that, girls who do not live with their families are more likely to experiment with smoking. Among them, the process of smoking initiates earlier than those who are living with their families.^13^ Besides, when friends smoke, boys are five times more likely to smoke, and this rate approximately increases to 9 times in girls.^13^ Therefore, sex-specific modeling of transition in different stages of smoking appears necessary.

In another point of view, few studies model the transition through smoking stages, which are limited to the single value probability to describe the transition process. Secondly, they are restricted to the certain statistical assumptions in the modeling process.^8,9,16^ On the other hand, the advantages of newly established model, are: (1) GMMs have less bias and error, (2) give more detailed information, and (3) produce findings that are more accurate.^17-19^ GMMs also relaxed some of the restricting statistical assumptions of the traditional models, so GMMs are feasible in practice.^19^

Moreover, due to sex differences,^13,20^ it seems that transition through smoking stages and the predictors should also be modeled sex-specifically. Based on a general search, no studies (if any) have been found that use sex-specific multi-group GMMs to examine the transition through smoking stages. This study aimed to use sex-specific multi-group GMMs to determine the transition through smoking stages and its predictors in adolescents in a longitudinal study.

## Materials and Methods

The study procedure details have been published elsewhere.^10,16^ This study pursued an innovative perspective to differentiate the girls and boys in the transition through smoking stages using the sex-specific GMMs.

### Study design

We used a cohort study to assess the transition through smoking stages of an illustrative sample of 10th-grade students in Tabriz metropolitan, Iran.

### Procedures

A total of 2959 (47.1%) girls and 2241 (42.9%) boys (4903 students) participated in this study, with 82 and 114 clusters (out of 865) respectively of male and female students randomly, and proportionally, selected using a multistage sampling procedure. The pupils filled out an anonymous multiple-choice questionnaire in the first and second year of study.

### Sample size

The sample size (of 4903) was much higher than the suggested rule-of-thumb of 10 samples per variable required for GMMs.^21^ Subsequently, the samples were allocated to schools and then classes proportional to the size of schools and classes, and separately for girls and boys.

### Variables and measures

The variables and measures description are presented elsewhere,^16^ though, we describe them here briefly. The participants’ demographic characteristics, including age, gender, living with parents, grade, major and socioeconomic status (SES) using a checklist. Also, the smoking-related variables, including having smoker friends (by a no/any binary response), having the smoker in the family (no/any), self-injury (no/any), and substance abuse (no/any), were measured using a validated checklist. We measured attitude toward smoking score by a 6-item questionnaire of Hill et al (in the range of -12 to 12)^22^ and self-esteem by a 10-item Rosenberg questionnaire.^23^ Also, we assessed general risk-taking behavior by one question (“Do you enjoy the exciting things that are a little risky?” with (0: No, 1: Yes) binary response). The main outcomes, including smoking status, intention to start smoking, and smoking during the past month/week, were assessed using an algorithm^16^ (see Table 1).

### Statistical analyses and model development: 

Data were analyzed by Mplus 7.4^18^ and SPSS 17 (SPSS Inc., Chicago, IL, USA) and expressed using mean (SD) and n(%) for numeric and categorical variables, respectively. The numeric variables were checked and confirmed for normality by distribution measures, namely skewness within ±1.5, and kurtosis within ± 2. Changes were assessed using McNemar and marginal homogeneity tests within the phases of the study.

We used growth mixture models (GMMs) to model the change of primary outcomes within two points of the study by estimating the intercept, showing the outcome level at zero time, and the slope, showing the change rate in outcome over time.^17,19^ Akaike information criteria (AIC), Bayesian information criteria (BIC), and adjusted BIC (ABIC) as well as the entropy values (>0.80) were utilized to select the optimal model were used to choose among the intercept only and 2- to 6-class linear models.^24,25^ Furthermore, we used the likelihood ratio test to compare the model of interest with a model with one smaller class.^24^ Finally, we fit the best-fitted GMMs with covariate to model the underlying demographic and smoking-related variables.^18^

## Results

### Participants’ profile

The details of the recruitment and participants’ profile are presented elsewhere.^10,16^ Of 4903 participants, 2956 (56.9%) were girls. The girls’ and boys’ mean age was 15.6 (SD 0.7) and 15.8 (SD 0.8) years, respectively. The girls had significantly higher grades than boys did, and the major of most girls were science and humanity. Most of the girls had a higher SES level compared to boys. The prevalence of cigarette smoking among girls was lower than among boys. The girls had a lower percent of the smoking friend and smokers in their family and had a lower prevalence of substance abuse than boys had (all *P* < 0.001). The girls had a lower attitude toward smoking and higher self-esteem scores than boys had (all *P* < 0.001).

Besides, for the primary outcomes of the study, the girls reported a higher percentage of “never smoked” in their smoking status. Also, the girls reported a higher percentage of “sure to never start smoking” in their smoking status than boys did. In contrast, the girls reported a lower percentage of “smoking during the past month” and “smoking during the past week” than boys did (all *P* < 0.001). Furthermore, there were no significant differences between girls and boys regarding other variables (Table 1).

### Distribution of changes in primary outcomes

There were 151 (5.9%) changes for girls vs. 300 (16.6%) changes for boys toward higher levels in smoking status (*P* < 0.001), and 141 (5.6%) changes for girls vs. 192 (10.4%) changes for boys toward higher intention to start smoking. However, there were 84 (3.3%) changes for girls vs. 126 (6.8%) changes for boys toward lower levels in intention to start smoking (*P* < 0.001). Furthermore, boys significantly tend to smoke more during the past month/week in the second assessment compared with the first assessment (both *P* < 0.05), but the changes were not significant among girls (both *P* > 0.05).

### Selecting the optimal GMMs class number

For the 2-class model, smaller AIC, BIC, and ABIC were observed compared with 1-class model, whereas the amount of change was negligible compared to the models with a higher number of classes. Besides, the entropy value was acceptable for this model (0.959). Therefore, we chose the optimal 2-class model for further assessments (Table 2).

### Name and the prevalence of the classes in the 2-class GMMs

**Figure 1:**
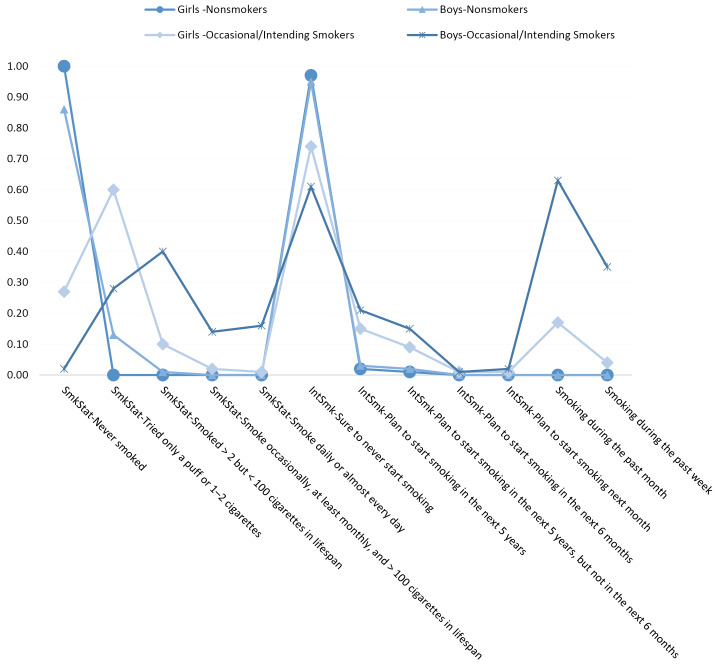
The loading of outcomes in the probability scale for two classes in girls (n=2956) and boys (n=2241). SmkStat: Smoking Status; IntSmk: Intention to start smoking.

Figure 1 presents the sex-specific loading of the 2-class model for the primary outcomes. Class 1 showed similar patterns between girls and boys for all outcomes, except for “Never smoked” and “Tried only a puff or 1–2 cigarettes” items, wherein light differences could be observed. Both girls and boys responded “Never smoked” and “Sure to never start smoking” and “not smoked” items with a significantly high probability (all *P* < 0.05). We named this class *“Non-smokers,”* which had a prevalence of 48.7% among girls vs. 34.4% among boys.

Class 2 showed different patterns between girls and boys for nearly all outcomes. For girls, the smoking status had significant loadings on lower and middle levels. However, boys had significant loadings on the middle and higher levels of the items. Among the girls, the “never smoked,” “Tried only a puff or 1–2 cigarettes,” and “Smoked > two but < 100 cigarettes in lifespan” items, while among the boys, “Tried only a puff or 1–2 cigarettes” and “Smoked > two but < 100 cigarettes in lifespan” and “Smoke daily or almost every day” items had significantly high loadings. Besides, the loadings of “not smoked” items for smoking during the past week were significant for girls, while the loading of both “Not smoked” and “Smoked” items was significant for boys (All *P* < 0.05). On the other hand, in class 2, the same pattern was observed for girls and boys for intention toward smoking and smoking during the past month, with significant loadings on nearly all items for both girls and boys (all *P* < 0.05). The values of loadings on “Smoked” item were considerably larger among boys than that among girls. We named this class “Occasional/Intending smokers”, which had a prevalence of 8.1% among girls vs. 8.8% among boys, with a similar pattern for both assessment times (Figure 1).

### Change in primary outcomes based on GMMs

For all outcomes, in both classes, the intercepts of the model were negative and significant compared with the intercept of the “Occasional/Intending smokers” among boys (all *P* < 0.05), indicating a lower level of the outcomes compared with the reference level.

Besides, the slope of the model was positive and significant for all outcomes in both “Non-smokers” and “Occasional/Intending smokers”, indicating an increased rate of change in the outcomes over time. There was an exception for smoking during the past month/week among girls in “Non-smokers”, wherein a negative slope was observed (Table 3).

### Background characteristics and changes in outcomes

Higher SES, having non-mathematics majors, having a smoker friend, higher levels of attitude toward smoking, and general risk-taking behavior were directly related to positive changes in smoking status. Also, the results indicated that higher age, living without parents, higher SES, having non-mathematics majors, having a smoker friend, and higher levels of attitude toward smoking were directly related to positive changes in intention to start smoking (all *P* < 0.05). Besides, higher age, higher SES, having a mathematics major, having a smoker friend, having a smoker in family, and higher levels of attitude toward smoking were directly related to changes to smoking during the past month/week (all *P* < 0.05) (Table 4).

## Discussion

### A general overview of the most important results 

This study is one of the rare longitudinal studies that use sex-specific GMMs to describe the transition through smoking stages, and model the transition predictors. GMMs presented an optimal 2-class model, utilizing information criteria: the “Occasional/Intending smokers” with 8.1% and 8.8% and the “Non-smokers” with 48.7% and 34.4% prevalence among girls and boys, respectively. Remarkably, negative intercepts and the positive slopes (except for smoking during the past month/week among girls in the “Non-smokers”), indicated negative level and the increasing rate of change in the outcome categories over time, respectively. Besides importantly, age, living with parents, SES, major, having smoker friends, having a smoker in family, self-injury, substance abuse, attitude toward smoking, and general risk-taking behavior were significantly related changes in outcomes and affected the speed of transition. Also, significant differences were observed between girls and boys in terms of changes in smoking stages, intention to start smoking, smoking during the past month/week over time. To sum up, it can be concluded that GMMs provide relevant findings of the sex-specific transition through smoking stages and the predictors.

### Utilizing GMMs

GMMs comprise distinguishing features over conventional statistical methods that have been used to study the transition in smoking stages as a latent variable.^26^ GMMs, like traditional methods, classify the heterogeneous population into homogenous subgroups and construct latent classes. However, in our data, GMMs, by fitting a tailored transition model, provided more information than conventional methods. In contrast, the traditional models are restricted to a single probability of transition and do not produce the statistical significance for the effect of the covariates.^7,8,11,16^ Furthermore, GMMs are not limited to the restricting statistical assumptions for estimating the parameters, which may not be satisfied in many practical situations.^17-19^ These features propose GMMs in studies aiming at assessing transition through smoking stages, taking an advantage over the conventional procedures.^7-9,11,16^

### The process of sex-specific transition in smoking stages 

In general, an increasing trend of transition to higher stages of smoking over time was observed. In the “Non-smokers”, girls moved more rapidly towards higher levels of smoking than boys did in terms of smoking status and intention to start smoking, and the difference in smoking status was considerable. Also, there was a positive slope for smoking among boys during the past week/month, but this slope became negative among girls. In the “Occasional/Intending smokers”, the slopes of all outcomes were positive among both girls and boys, with a negligible difference. Other studies have suggested various possibilities for differences between girls and boys, which can be explained by the verity in smoking definitions and instruments, and stages of smoking. Nevertheless, the results of a longitudinal study, which examined the transition process in adolescents, are consistent with the results of the present study showing a positive trend in the transition through smoking stages, and the transition process is usually progressive, and return is unlikely.^7^

### The predictors of transition in smoking stages 

Transition through smoking stages accelerated by increasing in age, not living with parents, SES, having a smoker friends, having a smoker in family, self-injury, substance abuse, attitude toward smoking, and general risk-taking behavior. The major plays an interesting role so that non-mathematics students create a positive slope in terms of smoking status and intention to start smoking. However, in terms of smoking during the past week/month, non-mathematics students create a negative slope. Almost similar results have been reported.^7,9,11,13,16,27-29^ Of course, there were differences in the predictors and the size of the reported effects, which can be explained by various definitions of the outcomes and measurement tools, variety in participants, and dissimilarities in study settings.

### Strengths, limitations, and future directions

The present study’s main strengths are as follows: this is one of the few studies that present the transition process through smoking stages using sex-specific optimal GMMs. Moreover, the length of the study and the large sample size can be noted. On the other hand, the results should be interpreted, taking into account the existing limitations: First, the two measurements in the present study make it impossible to draw higher order models. Second, the results depend on the self-reported measurement tool, although similar studies have used the same approach.^7,27-29^ Third, the generalizability of the results is limited to the tenth-grade students of Tabriz. Fourth, time-independent covariates were utilized in the study baseline. Future studies are strongly recommended to explore these issues.

## Conclusions and Implications

In general, based on sex-specific optimal GMMs model, a sex-dependent transition through smoking stages was observed. On the other hand, predictors accelerated this rising trend, and there was a significant difference between girls and boys in terms of these predictors. The study highlighted the importance of stopping the initiation and preventing transition through smoking stages with special sex-specific planning for adolescent girls and boys. These programs should be designed with a focus on essential predictors of transition.

## Acknowledgements

We would like to appreciate the collaboration of Research Deputy of Tabriz University of Medical Sciences for their appreciated contribution in this study. This work was supported by the Research Deputy of Tabriz University of Medical Sciences [Grant no 5/D/509208].

## Funding

This work was supported by the Research Deputy of Tabriz University of Medical Sciences [Grant no 5/D/509208].

## Competing interests

Mohammad Asghari-Jafarabadi is an Associate Editor for Health Promotion Perspectives. Other authors declare that there is no conflict of interest.

## Ethics approval

The institutional review board of Tabriz University of Medical Sciences approved the protocol (ethics code: IR.TBAMED.REC.1396.448). The participants were free to participate in the study, and privacy was preserved. All participants filled and signed the informed consent and assent. We have complied with the World Medical Association Declaration of Helsinki regarding the ethical conduct of research involving human subjects.

## Authors’ contributions

All authors read and approved the final manuscript. AMA conceived of the study and participated in the design and data collection. MAJ, AMA, and NJ participated in data analyses and manuscript preparation.

**Table 1:** Participants’ profile

**Characteristics**	**Girls (n=2956)**		**Boys (n=2241)**		***P *** **value** ^a^
	**N/mean**	**%/SD**	**N/mean**	**%/SD**	
Age (y)	15.6	0.7	15.8	0.8	<0.001
Living with parents	2651	94.9%	1965	93.8%	0.089
Grade	17.24	1.78	15.45	2.01	<0.001
Socio-economic status					
Low	974	37.5%	859	44.0%	<0.001
Moderate	553	21.3%	364	18.6%	
High	1071	41.2%	729	37.3%	
Major						
Mathematics	649	22.0%	547	24.4%	<0.001
Sciences	927	31.4%	397	17.7%	
Humanities	531	18.0%	274	12.2%	
Technical	849	28.7%	1023	45.6%	
Smoking cigarette	390	16.5%	764	41.5%	<0.001
Having a smoker friend	245	8.8%	651	30.9%	<0.001
Having a smoker in the family	1020	36.9%	907	43.6%	<0.001
Self-injury	409	14.7%	338	16.3%	0.137
Substance abuse	17	0.6%	50	2.4%	<0.001
Attitude toward smoking					
-12	1728	61.9%	1126	53.6%	<0.001
-9 to -11	633	22.7%	486	23.1%	
>-9	432	15.5%	489	23.3%	
Self-esteem	18.16	4.97	17.36	4.51	<0.001
General risk-taking behavior	1615	58.0%	1234	59.0%	0.500
Smoking status					
Never smoked	2435	87.7%	1436	70.5%	<0.001
Tried only a puff or 1–2 cigarettes	290	10.5%	320	15.7%	
Smoked > 2 but < 100 cigarettes in lifespan	39	1.4%	152	7.5%	
Smoke occasionally, at least monthly, and > 100 cigarettes in a lifespan	5	0.2%	51	2.5%	
Smoke daily or almost every day	6	0.2%	77	3.8%	
Intention to start smoking					
Sure to never start smoking	2563	93.1%	1839	87.8%	<0.001
Plan to start smoking in the next five years	93	3.4%	152	7.3%	
Plan to start smoking in the next five years, but not in the next six months	70	2.5%	99	4.7%	
Plan to start smoking in the next 6 months	8	0.3%	2	0.1%	
Plan to start smoking next month	19	0.7%	2	0.1%	
Smoking during the past month	77	2.8%	284	13.5%	<0.001
Smoking during the past week	21	0.8%	154	7.3%	<0.001

^a^ *P* values from independent t test and χ2 test for numeric and categorical variables, respectively.

**Table 2:** Models’ information criteria and n (%) within classes

**Models**	**Log-likelihood**	**Free Parameters no**	**P-MLR**	**AIC**	**BIC**	**ABIC**	**entropy**	**n(%)_C1_G**	**n(%)_C1_B**	**n(%)_C2_G**	**n(%)_C2_B**	**n(%)_C3_G**	**n(%)_C3_B**	**n(%)_C4_G**	**n(%)_C4_B**	**n(%)_C5_G**	**n(%)_C5_B**	**n(%)_C6_G**	n(%)_C6_B
Intercept Only	-18340.4	81	-	36842.8	37373.7	37116.3	1.000												
1 Class	-18463.6	33	<0.001	36993.2	37209.5	37104.7	1.000	2950 (0.568)	2241 (0.432)										
2 Class	-15253.6	50	<0.001	30607.2	30934.9	30776.0	0.959	2528 (0.487)	1786 (0.344)	422(0.081)	455(0.088)								
3 Class	-14614.0	67	<0.001	29362.0	29801.2	29588.3	0.811	2533 (0.488)	1464 (0.282)	0.0 (0.000)	530 (0.102)	417 (0.080)	247 (0.048)						
4 Class	-14276.5	84	<0.001	28721.0	29271.6	29004.6	0.850	101 (0.019)	231 (0.045)	2384 (0.459)	1470 (0.283)	97 (0.019)	138 (0.027)	368 (0.071)	402 (0.077)				
5 Class	-14072.8	101	<0.001	28347.5	29009.5	28688.6	0.867	116 (0.022)	427 (0.082)	385 (0.074)	253 (0.048)	82 (0.016)	122 (0.024)	2298 (0.443)	1378 (0.266)	69 (0.013)	61 (0.012)		
6 Class	-14005.8	118	<0.001	28247.6	29021.0	28646.1	0.885	172 (0.033)	332 (0.064)	130 (0.025)	141 (0.027)	72 (0.014)	69 (0.013)	94 (0.018)	104 (0.020)	2289 (.441)	1472 (0.284)	193 (0.037)	123 (0.024)

AIC, Akaike information criteria; BIC, Bayesian information criteria; ABIC: adjusted Bayesian information criteria; C: Class. P-MLR: *P* value based on Lo–Mendell–Rubin test

**Table 3:** Model's intercepts and slopes for change in primary outcomes in three classes

	**Class 1** **(Non-smokers)**				**Class 2** **(Occasional/Intending smokers)**			
	**Girls** **(n=173)**		**Boys** **(n=243)**		**Girls** **(n=415)**		**Boys** **(n=521)**	
	**I**	**S**	**I**	**S**	**I**	**S**	**I**	**S**
Smoking status	-8.5	2.7	-5.5	0.6	-2.7	0.8	Referent	0.9
Intention to start smoking	-2.9	0.5	-2.4	0.4	-0.6	0.2	Referent	0.3
Smoking during the past month	-8.5	-0.9	-5.5	1.2	-2.2	0.3	Referent	0.2
Smoking during the past week	-6.6	-2.5	-5.7	1.4	-2.5	0.5	Referent	0.5

I: Intercept; S: Slope. All intercepts and slopes were significant (all *P* values<0.05).

**Table 4:** Relationship between changes in outcomes and participants' characteristics

**Characteristics**	**Outcomes**			
	**Smoking status**	**Intention to start smoking**	**Smoking during the past month**	**Smoking during the past week**
Age (y)	0.01	0.13	0.08	0.25
Living with parents	-0.06	-0.14	-0.30	-0.20
Grade	0.04	0.02	0.08	0.04
Socio-economic status				
Low	Referent	Referent	Referent	Referent
Moderate	-0.05	0.21	0.23	0.35
High	0.29	0.20	0.27	0.31
Major				
Mathematics	Referent	Referent	Referent	Referent
Sciences	0.35	0.31	-0.51	-0.79
Humanities	0.53	0.16	-0.18	-0.96
Technical	0.42	0.20	-0.38	-0.88
Having a smoker friend	0.19	0.31	0.31	0.57
Having a smoker in the family	0.04	0.09	0.33	0.20
Self-injury	0.07	-0.03	0.43	0.77
Substance abuse	0.06	-0.02	0.06	0.07
Attitude toward smoking				
-12	Referent	Referent	Referent	Referent
-9 to -11	0.10	0.23	0.50	0.10
> -9	-0.03	0.16	0.35	0.87
Self-esteem	0.02	-0.02	-0.03	0.04
General risk-taking behavior	0.25	0.05	0.22	-0.04

The estimates for significant results are shown in bold font. The estimates are provided based on the slope of the model showing changes in outcomes' categories.
